# MicroRNA-mediated host defense mechanisms against pathogens and herbivores in rice: balancing gains from genetic resistance with trade-offs to productivity potential

**DOI:** 10.1186/s12870-022-03723-5

**Published:** 2022-07-18

**Authors:** Kishor Kumar, Swarupa Nanda Mandal, Kumari Neelam, Benildo G. de los Reyes

**Affiliations:** 1 Faculty Centre for Integrated Rural Development and Management, Ramakrishna Mission Vivekananda Educational and Research Institute, Narendrapur, Kolkata, 700103 India; 2 Department of Plant and Soil Science, Texas Tech University, Lubbock, TX-79415 USA; 3 Department of Genetics and Plant Breeding, Bidhan Chandra Krishi Viswavidyalaya, Extended Campus, Burdwan, West Bengal 713101 India; 4 School of Agricultural Biotechnology, Punjab Agricultural University, Ludhiana, Punjab 141004 India

**Keywords:** Rice, MicroRNA, Plant immunity, Defense-yield trade-off, Genome editing

## Abstract

**Background:**

Rice (*Oryza sativa* L.) is the major source of daily caloric intake for more than 30% of the human population. However, the sustained productivity of this staple food crop is continuously threatened by various pathogens and herbivores. Breeding has been successful in utilizing various mechanisms of defense by gene pyramiding in elite cultivars, but the continuous resurgence of highly resistant races of pathogens and herbivores often overcomes the inherent capacity of host plant immunity. MicroRNAs (miRNAs) are endogenous, short, single-stranded, non-coding RNA molecules that regulate gene expression by sequence-specific cleavage of target mRNA or suppressing target mRNA translation. While miRNAs function as upstream regulators of plant growth, development, and host immunity, their direct effects on growth and development in the context of balancing defenses with agronomic potential have not been extensively discussed and explored as a more viable strategy in breeding for disease and pest resistant cultivars of rice with optimal agronomic potentials.

**Results:**

Using the available knowledge in rice and other model plants, this review examines the important roles of miRNAs in regulating host responses to various fungal, bacterial, and viral pathogens, and insect pests, in the context of gains and trade-offs to crop yield. Gains from R-gene-mediated resistance deployed in modern rice cultivars are often undermined by the rapid breakdown of resistance, negative pleiotropic effects, and linkage drags with undesirable traits. In stark contrast, several classes of miRNAs are known to efficiently balance the positive gains from host immunity without significant costs in terms of losses in agronomic potentials (i.e.*,* yield penalty) in rice. Defense-related miRNAs such as *Osa-miR156*, *Osa-miR159*, *Osa-miR162*, *Osa-miR396*, *Osa-530, Osa-miR1432, Osa-miR1871,* and *Osa-miR1873* are critical in fine-tuning and integrating immune responses with physiological processes that are necessary to the maintenance of grain yield. Recent research has shown that many defense-related miRNAs regulate complex and agronomically important traits.

**Conclusions:**

Identification of novel immune-responsive miRNAs that orchestrate physiological processes critical to the full expression of agronomic potential will facilitate the stacking of optimal combinations of miRNA-encoding genes to develop high-yielding cultivars with durable resistance to disease and insect pests with minimal penalties to yield.

## Background

The world population is increasing at an alarming rate, with projections of 10 billion by 2050 [1]. At least 60% more food grains will have to be produced to ensure that the global needs for staple foods are secured in the twenty-first century and beyond [1]. Rice (*Oryza sativa* L.) is one of the major staple food crops that provide calories to more than one-third of the human population. Rice productivity is constantly challenged by the negative impacts of pathogens, insect herbivores, and other parasites. These biotic stresses, particularly pathogens, account for 20–30% of losses in global rice yields [2].

Pathogens and their host plants continuously compete for dominance in a co-evolutionary battle. Plants have evolved multi-layered defense strategies against pathogen invasion [3, 4]. Host plants induce complex defense mechanisms by activating or suppressing a large array of genes in response to pathogen attacks [5]. Such mechanisms are facilitated by the ability of a host plant to recognize a myriad of pathogen-associated molecular patterns (PAMPs) or damage-associated molecular patterns (DAMPs) during the initial stages of pathogen or herbivore invasion [6]. The pathogen-triggered PAMP or DAMP is usually recognized by pattern-recognition receptors (PRRs) on the surface of host cells. The PAMPs or DAMPs, in turn, activate the PAMP-triggered immunity (PTI) response by inducing many types of defense-related genes [7, 8].

Host-plant immunity is dependent on the successful activation of defense-related genes. Successful virulent pathogens often overcome the PTI by mediating effector-triggered susceptibility (ETS), which leads to disease development [9]. In response, host plants develop a secondary immune response known as effector-triggered immunity (ETI), mediated by intracellular receptor proteins encoded by R-genes [10]. The product of R-genes (R-proteins) binds to specific pathogen effectors producing a more complex and robust hypersensitive response (HR), which further mediates cell death to restrict the growth of the pathogen at the sites of infection [3]. In rice, many R-genes against pathogens and insect pests have been identified and characterized. Most of these R-genes are effectively utilized to enhance resistance through introgression breeding. However, their efficacies are often overcome within a certain period of time due to the evolution of new resistant races that can no longer be recognized by the R-gene products. Moreover, unregulated expression of R-genes imposes a substantial demand on cellular resources, which negatively affects plant growth with trade-offs to productivity in terms of penalty to grain yield [11–13]. Therefore, in addition to R-genes, exploring other types of genetic defenses, including those that are mediated by other regulatory molecules such as microRNAs (miRNAs) is a potentially important strategy for balancing efficient disease and pest management with sustainable rice production.

MicroRNAs (miRNAs) are short (~ 22 nucleotides), endogenous, single-stranded, non-coding RNAs that form a characteristic stem-loop structure. Their function is to negatively regulate the expression of their target genes (i.e., usually a regulatory gene such as a transcription factor) at the post-transcriptional level. Importantly, microRNAs act as crucial modulators of various cellular and biological processes, including plant growth, development, reproduction, and responses to biotic and abiotic stresses [14–16]. Plant miRNAs are transcribed as primary miRNA (pri-miRNA) from miRNA-encoding genomic loci (*MIR* loci) by RNA polymerase II [17]. The long stem of the looped pri-miRNAs is subsequently processed by RNAse III enzymes called DICER-like 1 (DCL1) proteins in association with *hyponastic leaves 1* (HYL1) and *serrate* (SE) into double-stranded miRNA-miRNA* (*passenger strand of miRNA) duplex [18, 19]. The miRNA/miRNA* duplex is transported from the nucleus into the cytoplasm and subsequently methylated by HUA ENHANCER 1 (HEN1) to prevent degradation [20]. The guide miRNA is loaded into ARGONAUTE1 (AGO1) to form a functional RNA-induced silencing complex (RISC) [21]. Complementarity between the silencer miRNA and its target transcript allows the RISC complex to trigger complete inhibition of protein synthesis either through the degradation of the mRNA or inhibition of its translation.

**Fig. 1:**
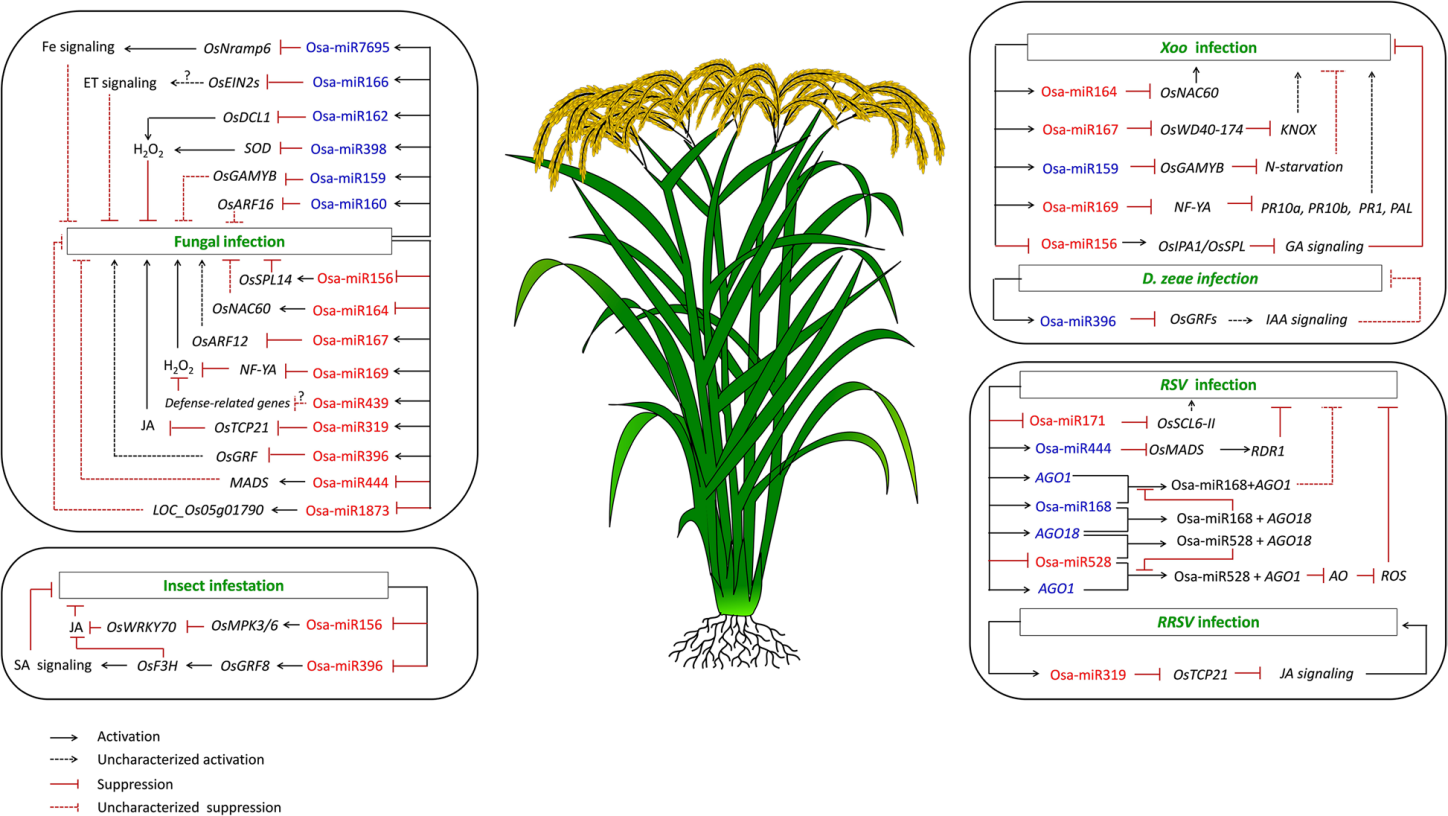
Summary of known miRNA-mediated defense mechanisms against pathogens and herbivores in rice. The left panel indicates an overview of the miRNAs responsive to fungal pathogens and insects. The right panel shows the miRNAs involved in resistance to bacterial and viral diseases. The miRNAs written in blue and red represent the positively and negatively regulated miRNAs, respectively. *Xoo- Xanthomonas oryzae* pv. *oryzae; D. zeae- Dickey Zeae,* RSV- Rice stripe virus; RRSV- Rice ragged stunt virus

In the past few years, the rapid development of next-generation sequencing (NGS) technologies and powerful algorithms for the prediction and modelling of interactions at the genetic, genomic, and molecular levels opened new paths in miRNA discovery. Molecular genetic approaches like 5’RACE, degradome sequencing, stem-loop RT-PCR, reporter gene analysis, loss- or gain-of-function mutation experiments have led to the discovery and unraveling of the function of the large number of miRNAs [22]. In rice, numerous miRNAs that fine-tune host immune response and also those that regulate plant growth have been identified, cloned, and functionally validated through loss-of-function or gain-of-function mutation experiments. In this mini-review, we specifically focus on the regulatory impacts of functionally characterized defense-related miRNAs in rice and their roles in fine-tuning complex traits of agronomic importance. Cautionary aspects of innovative strategies for miRNA manipulation towards understanding the balance and trade-offs between defense and productivity in terms of yield are discussed. In the subsequent sections, we highlight the role of miRNAs in mediating resistance to various pathogens and herbivores (Fig. 1, Table 1).

**Table 1:** Comprehensive list of miRNAs involved in rice immunity against pathogens and herbivores

Organisms	MicroRNAs	Target genes	Regulation	Pathogens/ herbivores	References
Fungus	*Osa-miR156fhl-3p*	*OsSPL14*	Negative	*M. oryzae*	[23]
	*Osa-miR159a*	*OsGAMYB*	Positive	*M. oryzae*	[24]
	*Osa-miR160a*	*ARF16*	Positive	*M. oryzae*	[25]
	*Osa-miR162a*	*OsDCL1*	Positive	*M. oryzae*	[26]
	*Osa-miR164a*	*OsNAC60*	Negative	*M. oryzae,* *R. solani*	[27]
	*Osa-miR166k-166 h*	*EIN 2*	Positive	*M. oryzae,* *F. fujikuroi*	[28]
	*Osa-miR167d*	*ARF12*	Negative	*M. oryzae*	[29]
	*Osa-miR169*	*NF-YA*	Negative	*M. oryzae*	[30]
	*Osa-miR319*	*OsTCP21*	Negative	*M. oryzae*	[31]
	*Osa-miR396*	*OsGRFs*	Negative	*M. oryzae*	[32]
	*Osa-miR398b*	*SOD*	Positive	*M. oryzae*	[33]
	*Osa-miR439a*	*Defense-related genes*	Negative	*M. oryzae*	[34]
	*Osa-miR444*	*MADS*	Negative	*M. oryzae*	[35]
	*Osa-miR530*	*HDFR-TS*	Negative	*M. oryzae*	[36]
	*Osa-miR1432*	*OsEFH1*	Negative	*M. oryzae*	[37]
	*Osa-miR1871*	*OsMFAP1*	Negative	*M. oryzae*	[38]
	*Osa-miR1873*	*LOC_Os05g01790*	Negative	*M. oryzae*	[39]
	*Osa-miR7695*	*OsNramp6*	Positive	*M. oryzae*	[40]
Bacteria	*Osa-miR156*	*OsSPLs*	Negative	*X. oryzae*	[41]
	*Osa-miR159b*	*OsGAMYB*	Positive	*X. oryzae*	[42]
	*Osa-miR164a*	*OsNAC60*	Negative	*X. oryzae*	[42]
	*Osa-miR167d-5p*	*OsWD40–174*	Negative	*X. oryzae*	[42]
	*Osa-miR169o*	*NF-YA*	Negative	*X. oryzae*	[43]
	*Osa-miR396f*	*OsGRFs*	Positive	*Dickeya zeae*	[44]
Viruses	*Osa-miR168*	*AGO1*	Positive	RSV	[45]
	*Osa-miR171b*	*OsSCL6-II*	Negative	RSV	[46]
	*Osa-miR319*	OsTCP21	Negative	RRSV	[47]
	*Osa-miR444*	*MADS Box*	Positive	RSV	[48]
	*Osa-miR528*	*L-ascorbate oxidase*	Negative	RSV	[49, 50]
Herbivores	*Osa-miR156*	*OsMPKs*	Negative	BPH	[51]
	*Osa-miR396*	*OsGRF8*	Negative	BPH	[52]

OsSPL- SQUAMOSA promoter-binding protein-like transcription factor; ARF- Auxin responsive factor; DCL- Dicer-like, EIN- Ethylene insensitive; NF-YA- Nuclear Factor Y-A; OsTCP- Teosinte branched/Cycloidea/Proliferating cell factor; OsGRF- Growth regulating factors; SOD- Superoxide dismutase, DHFR-TS - Dihydrofolate reductase/thymidylate synthase; OsEFH1- EF-hand family protein 1; OsMFAP1- Microfibrillar-associated protein 1; OsNramp- Natural resistance-associated macrophage pathogen, AGO- ARGONAUTE 1; OsSCL- Scarecrow-like; RSV- Rice stripe virus; RRSV- Rice ragged stunt virus; BPH-Brown planthopper

### MicroRNAs against fungal pathogens

**Fig. 2:**
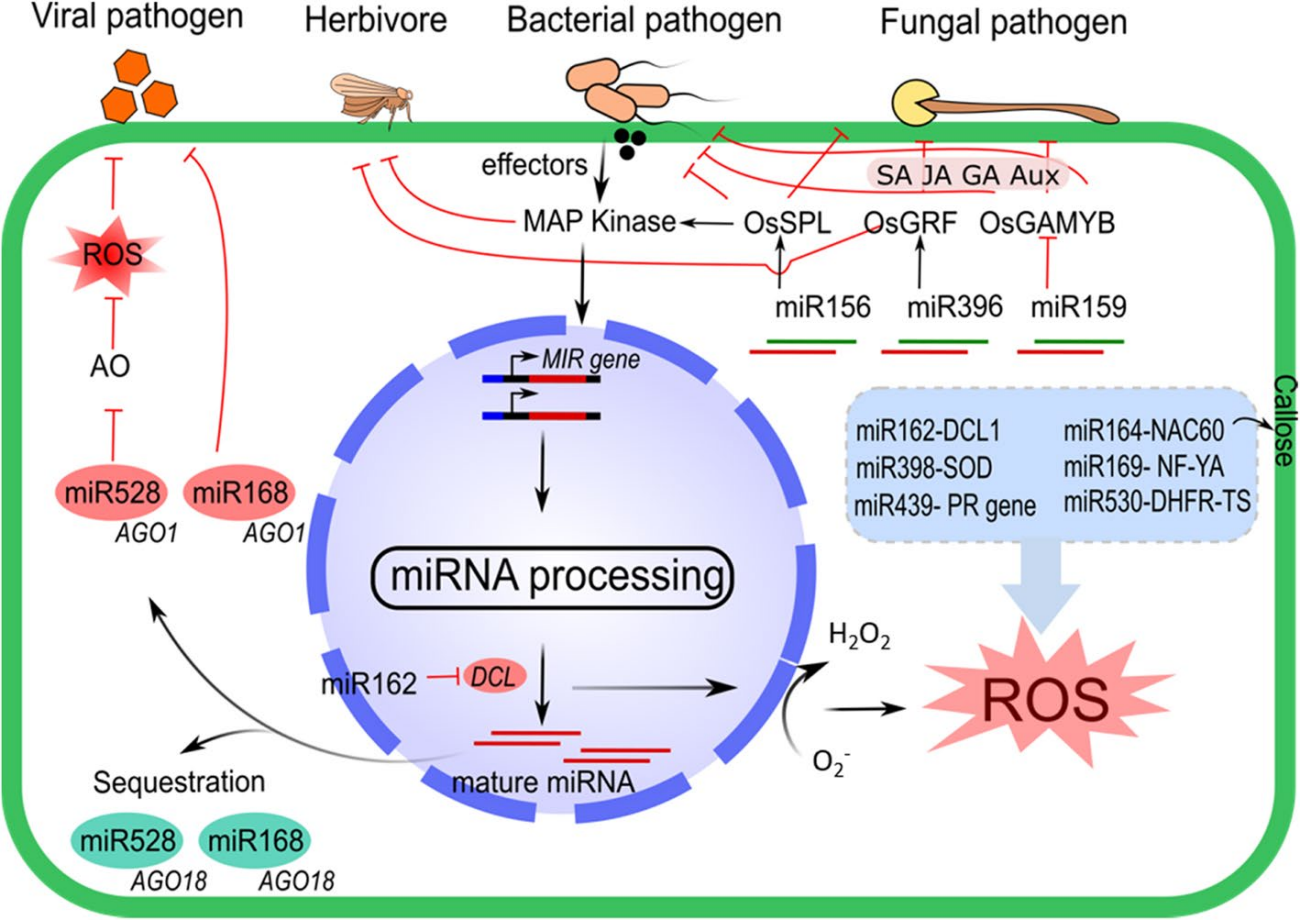
Functionally characterized miRNAs associated with the immune response against bacterial, fungal, and viral pathogens, as well as insect herbivores. Pathogen-derived effector molecules elicit the expression of MIR genes by RNA polymerase II via mitogen-activated protein (MAP) kinase signaling. The long hairpin transcript is processed by Dicer-like protein (DCL). The *miR162* targets the DCL and produces ROS. Various miRNAs such as *miR162*, *miR164*, *miR169*, *miR398*, and *miR439* also participate in the immune response mechanism via ROS. On the other hand, a tug of war between AGO1 and AGO18 for binding to *miR528* and *miR168* facilitates the expression of strong resistance against the invading viral pathogen through the production of reactive oxygen species (ROS). The *miR156*, *miR396*, and *miR159* confer resistance to pathogens and insect pests by targeting transcription factors through a mechanism modulated by phytohormones

In rice, the two-layered immune system (PTI and ETI) has been shown to play important roles in defense against fungal pathogens such as *Magnaporthe oryzae* [53]. Pattern Recognition Receptors (PRRs) such as CEBiP, LYP4, and LYP6 are known to recognize the pathogen-associated molecular patterns (PAMP) and induce the PAMP-triggered immunity [54, 55]. Whereas the products of R-genes recognize divergent pathogen effectors that activate effector-triggered immunity (ETI), their functionality for recognizing the target effectors depends on several structural features. However, resistance conferred by a single R-gene is quickly overcome by the emergence of new pathotypes that can evade the effects of the R-gene products. Therefore, pyramiding multiple R-genes in the same genetic background represents a more robust approach to develop rice cultivars with broad-spectrum resistance. The caveat to this approach is that R-gene pyramiding by conventional breeding (even with a marker-assisted approach) requires multiple rounds of hybridization and selection that are often confounded by the negative effects of linkage drags. A random combination of multiple R-genes in the same genetic background may not always produce positive or optimal effects, thus the trade-off effects between resistance and yield have been a major challenge in maximizing and optimizing the gains from such an approach for yield improvement. The use of miRNAs provides an alternative strategy to develop broad-spectrum resistance against fungal pathogens in rice cultivars. The increasing number of evidence support that miRNAs also regulate the ETI and PTI [56, 57]. In particular, it has been shown that certain miRNAs fine-tune the expression of innate immunity in certain cultivars through the integration of R-gene regulation, hormone signaling, callose deposition, and production of reactive oxygen species (ROS) such as superoxide radicals (O^•^_2_^−^), hydroxyl radicals (OH^·^) and hydrogen peroxide (H_2_O_2_) (Fig. 2).

#### MicroRNAs as positive regulators of immunity against blast disease in rice

Several miRNAs, including *Osa-miR159*, *Osa-miR160, Osa-miR162a, Osa-miR166k, Osa-miR166h, Osa-miR398b,* and *Osa-miR7695* have been shown to function as positive regulators of defenses against the rice blast disease caused by fungus *Magnaporthe oryzae* [26]. For instance, the *Osa-miR159a* fine-tunes host plant growth and immunity by inhibiting its three target genes, *OsGAMYB*, *OsGAMYBL*, and *OsZF*. The *OsGAMYB* and *OsGAMYBL* are transcriptional regulators of gibberellic acid signaling, while *OsZF* is a C3HC4- domain-containing zinc finger protein involved in ubiquitin-protein transferase activity. Transgenic rice plants overexpressing a short tandem target mimic (STTM) to inhibit the *Osa-miR159a* showed enhanced host susceptibility to the disease. In contrast, the knock-down mutation of the target genes conferred resistance to *M. oryzae*.

In addition, the *Osa-miR159-GAMYB* module orchestrates the reproductive developmental process by its direct role in the regulation of flower, pollen, and seed development [24]. It is apparent that *Osa-159a* must be precisely spatiotemporally regulated to coordinate plant development and immunity. Similarly, leaf and seed development in rice has been shown to be regulated by *Osa-miR160* via auxin signaling, which also acts as a positive regulator of rice immunity against *M. oryzae.* Transgenic plants overexpressing *Osa-miR160* displayed significantly stronger resistance to blast disease. The *Osa-miR160a* enhances resistance to blast by suppressing the *AUXIN RESPONSE FACTOR 16* (*ARF16*), which in turn silences the indole-3-acetic acid (IAA) signaling [25]. Diverse cellular and physiological processes in plants are controlled by *miR166,* which belongs to a highly conserved family of miRNA molecules. The *miR166* targets the class III homeodomain-leucine zipper family (*HD ZIP III*) of transcription factors [58, 59]. In rice specifically, overexpression of the polycistronic miRNA containing two of the *miR166-*family members, i.e.*, Osa-miR166k* and *Osa-miR166h*, has been shown to cause stronger immunity against *M. oryzae* and *Fusarium fujikuroi,* through targeting of the *ethylene insensitive 2* (*EIN 2*) gene via cross-regulation. This mechanism has been shown to occur without negative effects on plant growth [28].

Plants produce H_2_O_2_ in response to biotic and abiotic stresses. It has been shown that *Osa-miR162a* fine-tunes the host’s innate immunity against *M. oryzae* by targeting the *Dicer-like 1* (*OsDCL1*) gene through the accumulation of intracellular H_2_O_2_ thereby facilitating cell death at the infection site. This mechanism also regulates other physiological processes that are critical to yield maintenance. Overexpression of *Osa-miR162a* showed enhanced resistance to *M. oryzae* by positively regulating many other defense-related genes [26]. Growing evidence suggests that loss-of-function of *OsDCL1* showed developmental defects in rice at the seedling stage, including dwarfism, root and shoot abnormality, and wilting of leaves. All of these effects indicate that *Osa-miR162a* optimizes growth and immunity without any yield penalty. ROS homeostasis is regulated by several key enzymes, including superoxide dismutase (SOD). Consistent with earlier reports, elevated levels of cellular H_2_O_2_ has been observed with the suppression of the Cu/Zn *superoxide dismutase* (*SOD*) genes by the *Osa-miR398b.* This process positively regulates host immunity against blast by negatively regulating the components of ROS production and homeostasis [25]*.*

Upon *M. oryzae* infection, higher activity of SOD is associated with accumulation of H_2_O_2_, which leads to enhanced resistance to blast [33]. ROS is also produced by an excess of iron (Fe), an essential micro-element required for photosynthesis and chloroplast maintenance. This process is critically regulated upon pathogen infection as the host and pathogen compete for available Fe. Recently, *Osa-miR7695* has been shown to function as a positive regulator of rice immune response by mediating the trade-off between defense and iron homeostasis. Rice plants overexpressing *Osa-miR7695* showed increased resistance to *M. oryzae* and stronger Fe accumulation at the site of infection. It was proposed that upon infection with *M. oryzae*, expression of *Osa-miR7695* leads to the suppression of the target gene *OsNramp 6 (Natural resistance-associated Macrophage Protein 6)*, which encodes an iron transporter [40, 60].

#### Negative regulation of defenses against rice blast by miRNAs

The *Osa-miR156fhl-3p*, *Osa-miR164a*, *Osa-miR167d*, *Osa-miR169a*, *Osa-miR319*, *Osa-miR396*, *Osa-439a, Osa-miR444*, *Osa-miR530, Osa-miR1432,* and *Osa-miR1873* have been identified as negative regulators of rice innate immunity against *M. oryzae.* These miRNAs have been reported to control rice innate immunity by targeting critical transcription factors. The *miR156* belongs to a conserved family that regulates plant growth, development, and yield by targeting the *SQUAMOSA promoter-binding protein-like transcription factor 14* (*SPL14*) and *WRKY45* transcription factor. In rice, overexpression of *Osa-miR156fhl-3p* in a target mimic mutant showed stronger resistance to blast by virtue of enhanced expression of the target genes *SPL14* and *WRKY45* transcription factors [23]. Expression of the *OsNAC60 transcription factor* is negatively regulated by the suppression of *Osa-miR164a* upon *M. oryzae* infection, which leads to the enhancement of defense responses [27]. Abolition of the entire *Osa-miR164a/OsNAC60* regulatory module has been shown to result in a susceptible phenotype. The *Osa-miR164a* has also been involved in controlling the sheath blight-causing fungus *Rhizoctonia solani* by activating the salicylic acid (SA) signaling pathway and expression of associated defense-related genes. Expression of *Osa-miR169a* has been shown to condition a strong resistance against *M. oryzae* by suppressing its target gene *nuclear factor Y-A* (*NF-YA*). Significant accumulation of *Osa-miR169a* has been documented in a susceptible genotype of rice with a somewhat decreased resistance level. Transgenic rice plants overexpressing *Osa-miR169a* have been shown to exhibit a higher level of susceptibility, which was associated with the downregulation of target genes and reduced accumulation of intracellular H_2_O_2_ [30]. The *Osa-miR530* controls H_2_O_2_ production by regulating the expression of dihydrofolate reductase/thymidylate synthase (DHFR-TS). It is noted that DHFR-TS participates in the maintenance of redox balance by producing the nicotinamide adenine dinucleotide phosphate (NADPH) and ROS. Subsequently, ROS is converted into H_2_O_2_ by SOD. Perturbation of *Osa-miR530* enhances resistance to blast disease through its effects on H_2_O_2_ accumulation. It is important to mention that blocking the *Osa-miR530* also positively affects flowering and seed maturation [36]. The *Osa-miR439a* negatively affects immunity by inhibiting the expression of defense-related genes and H_2_O_2_ production Suppression of the *Osa-miR439a* using target mimic mutants has been shown to compromise susceptibility to *M. oryzae* by induction of H_2_O_2_ [34].

The *Osa-miR319* modulates host immune response in the rice-*M. oryzae* interaction in a negative manner. Accumulation of *Osa-miR319* has been observed in susceptible genotypes as indicated by the suppression of the target gene *OsTCP21* upon *M. oryzae* infection. Blocking the conversion of a-linoleic acid (LnA) to hydroperoxy-octadecadienoic acid (HPODE) has been shown to inhibit the jasmonic acid (JA) signaling pathway [31]. The *Osa-miR444b.2* has also been identified among the many negative regulators of rice immunity against *M. oryzae*. Overexpression of *Osa-miR444b.2* enhances susceptibility to *M. oryzae* but with little impact on its target transcription factors *MADS27b* and *MADS57*. Altered expression of the target mimicry of *Osa-miR444b.2* resulted in enhanced resistance to blast [35]. The *Osa-miR444a* has been shown to positively regulate immunity against the *rice stripe virus* (RSV) by upregulating *OsRDR1* expression, which is facilitated by suppressing target *MADS*-*box* genes [48].

The *Osa-miR1432* has been shown to fine-tune resistance to disease as well as yield potential by targeting the *OsEFH1* (EF-hand family protein 1). Overexpression of *Osa-miR1432* has been shown to compromise resistance to *M. oryzae* with concomitant adverse effects on yield. On the other hand, inhibition of *Osa-miR1432* expression has enhanced resistance with positive effects on yield, attributed to the enhancement of PTI responses [37]. During *M. oryzae* infection, immunity and yield in rice are also balanced by the *Osa-miR1871*. Inhibition of *Osa-miR1871* expression greatly enhances resistance and yield, by virtue of the effects on the target*OsMFAP1* (Microfibrillar-associated protein 1) gene. In contrast, overexpression of *Osa-miR1871* had been shown to cause susceptibility to blast, with concomitant negative effects on grain yield [38].

The *Osa-miR1873* appeared to regulate resistance to rice blast in a negative manner. It has been shown that expression of *Osa-miR1873* leads to disease susceptibility through the suppression of a target gene with an unknown function (i.e., LOC_Os05g01790*,* which encodes a DUF868 domain-containing protein). Suppression of *Osa-miR1873* through the expression of the target mimicry mutant has been shown to enhance defense response against blast by fine-tuning host plant immunity and growth [39]. The *Osa-miR167d* modulates plant developmental and stress responses by targeting *auxin response factor* (*ARF*) genes. The *Osa-miR167d* also seemed to play a negative role in rice immunity against *M. oryzae* by blocking *ARF12* genes. Suppression of *Osa-miR167d* by overexpressing a target disease mimic mutant led to enhanced resistance to blast [29]. Characterization of *Osa-miR396* revealed its negative effect on defense response against *M. oryzae* by silencing multiple *OsGRF* genes. Overexpression of *Osa-miR396* promotes susceptibility to blast through the downregulation of *OsGRFs* genes. Suppression of *Osa-miR396* by expressing the target disease mimics mutant enhanced resistance to blast with concomitant improvement in yield [32].

### MicroRNAs regulating responses to bacterial pathogens

Five classes of miRNAs have been shown to modulate resistance against bacterial blight (BB) caused by *Xanthomonas oryzae* pv. *oryzae* (*Xoo*). Another class of miRNAs has been shown to positively regulate resistance against the bacterial foot rot disease caused by *Dickeya zeae*. Of the miRNAs involved in responses to BB, the *Osa-miR159b* has been shown to function as a positive regulator, while the others function as negative regulators. The *Osa-miR159b* enhances resistance to BB by repressing its target transcription factor *OsGAMYB* involved in gibberellic acid (GA) signaling. This process leads to negative effects on nitrogen assimilation as an outcome of the repression of the GA signaling pathway [42].

The *Osa-miR167* has also been shown to be involved in rice immunity to bacterial pathogens [57, 61]. For instance, *Osa-miR167d* abates immunity upon *Xanthomonas oryzae* pv. *oryzae* (*Xoo)* infection by suppressing the target gene *OsWD40–174,* which downregulates the lignin biosynthetic gene *OsKNOX* during leaf development [42]. Downregulation of KNOX hinders lignin biosynthesis [62]. Transgenic plants overexpressing *Osa-miR164a* showed enhanced susceptibility to *Xoo* by virtue of the downregulation of the *OsNAC60* transcription factor [42]. The role of the *miR164a/OsNAC60* regulatory module in blast resistance has been discussed earlier in this review. The *Osa-miR169o* represents the coordinated crosstalk regulation between BB and Nitrogen-use efficiency (NUE) in rice. Overexpression of the *miR169o* enhances NUE and promotes susceptibility to BB by suppressing the *nuclear factor Y-A* (*NF-YA*), which in turn causes significant downregulation of several defense-related genes, including *PR10b, PR1b, PR10a,* and PAL [43]. The *Osa-miR156* negatively regulates immunity against bacterial blight [41]. Transgenic plants overexpressing the target gene *IPA1* (*Ideal Plant Architecture1*) under the control of a pathogen-inducible promoter of *OsHEN1* have been shown to enhance both disease resistance and yield.

More recently, *Osa-miR396f* was shown to play a positive role in conferring resistance against bacterial foot rot disease caused by *Dickeya zeae.* Overexpression of *Osa-miR396f* in the susceptible rice variety Nipponbare showed enhanced immunity to *D. zeae* by suppressing the target gene *OsGRFs* (*Growth-Regulating Factors*). However, the precise molecular mechanism underpinning the immunity against bacterial foot roots mounted by *OsGRFs* is still unknown [44].

### MicroRNAs control resistance to viral pathogens

The miRNA-mediated gene regulation has emerged as a novel strategy to enhance antiviral defenses in crop plants, including rice [63]. Plants have evolved different RNA silencing mechanisms to respond to diverse classes of viral infections. Antiviral RNA silencing is triggered by virus-derived double-stranded RNA (dsRNA) that are directly recognized and processed by the host’s dicer-like proteins (DCL) to form virus-derived small-interfering RNAs (vsiRNAs) [64]. The vsiRNAs are incorporated into the ARGONAUTE (AGO) proteins that form the core component of the RNA-induced silencing complex (RISC). The functional RISC either cleaves the viral RNA or arrests the viral protein translation. Single-stranded viral RNAs require endogenous RNA-dependent RNA polymerases (RDRs) to synthesize dsRNA that serves as the substrate for DCLs to produce secondary vsiRNAs. To counteract the defense response of host plants, most viruses have developed specialized proteins known as viral suppressors of RNA silencing (VSRs) that impede the antiviral RNA silencing pathway and suppress the defense response [65].

MicroRNAs provide an additional layer of intrinsic defense against viral attacks. In rice, the *Osa-miR528* is well characterized and known to be involved in regulating defenses against the rice stripe virus (RSV). In cleavage-defective AGO18 mutants of rice, the *Osa-miR528* is sequestered away from AGO1, upon RSV infection. These, in turn, prevent the formation of a functional RNA-induced silencing complex. These events further lead to the enhanced expression of downstream gene AO, encoding an L-ascorbate oxidase and functions in the initiation of defense response against RSV through the accumulation of ROS [49]. Further research suggested that the transcription factor *OsSPL9* specifically regulates the expression of the *Osa-miR528-AO* module by binding to the promoter of the *Osa-miR528*. Mutation of *OsSPL9* has been shown to cause the dramatic downregulation of *Osa-miR528*, thereby inducing AO expression that leads to enhanced resistance to RSV [50].

The AGO18 also sequesters the *Osa-miR168* upon RSV infection. It has been shown that AGO18 competes with AGO1 for binding to *Osa-miR168,* resulting in an elevated level of AGO1-mediated resistance [45]. RSV infection triggers the expression of *Osa-miR444*, which positively regulates immune response. Transgenic plants overexpressing the *Osa-miR444* displayed a broad-spectrum resistance to RSV by inducing *OsRDR1* and by silencing several *MADS-box* transcription factors [48].

RSV infection of host rice plants perturbs the expression of *Osa-miR171.* Such impaired expression has been shown to severely affect plant height and chlorophyll content, thereby causing RSV-like symptoms. It has been demonstrated that plants expressing *miR171b* were less susceptible to RSV characterized by attenuated RSV symptoms [46]. Similarly, the *Osa-miR319* acts as a negative regulator of immunity against the ragged stunt virus (RRSV) in rice. Transgenic plants overexpressing the *Osa-miR319* have exhibited severe disease-like symptoms by negatively affecting the target gene *OsTCP21* (*TEOSINTE BRANCHED/CYCLOIDEA/PCF*) and subsequently suppressing JA-mediated plant defense pathways [47].

### MicroRNAs for insect resistance

Rice is host to more than 200 insect pests at different stages of its life cycle. Advances in genomics have greatly expanded the understanding of the role of miRNAs in the regulation of immune response to insect pests. During brown planthopper (BPH) infestation of rice, differentially expressed miRNAs have been identified by deep sequencing in an introgression line carrying the resistance gene *Bph15* in comparison to a susceptible genotype. Several known miRNAs, along with 183 other novel BPH-responsive miRNAs, have been identified to be involved in the regulation of either basal defenses or targeted resistance to BPH [66]. Similarly, integrated analysis of miRNA and mRNA expression profiles identified numerous BPH-responsive miRNAs that are differentially expressed between transgenic lines carrying the resistance gene *Bph6* and wild-type [67]. *Osa-miR156* was found to function as a negative regulator of the BPH resistance. Sequestration of the *Osa-miR156* using a target mimic mutant of *Osa-miR156* displayed enhanced resistance to BPH by positively regulating the *OsMPK3* and *OsMPK6* genes. The transcript level of *OsWRKY70* transcription factor was found to be significantly reduced in the *Osa-miR156*-sequestered target mimic mutant that represses jasmonic acid (JA) biosynthesis and signaling [51]. Another miRNA, *Osa-miR396,* also acts as a negative regulator of BPH resistance. Downregulation of *Osa-miR396* enhances the expression of the *growth-regulating factor 8* (*OsGRF8*) gene, which in turn positively regulates the *flavanone 3-hydroxylase* (*OsF3H*) gene involved in the flavonoid biosynthesis. The *OsF3H* positively regulates the BPH resistance through its positive effects on the salicylic acid (SA) pathway and negative effects on the jasmonic acid (JA) pathway [52]*.*

### MicroRNA-mediated regulation of R-genes: lessons from rice and other models

Modern cultivars are being developed by pyramiding multiple R-genes as well as other types of genes involved in quantitative mechanisms as a major strategy to avoid substantial losses to crop yield due to diseases and pests. However, it has been shown that constitutive expression of R-genes often imposes high fitness costs, mainly with deleterious consequences to plant growth and development. Therefore, the expression of R-genes must be optimized in the right spatial and temporal manner to minimize trade-offs.

**Fig. 3:**
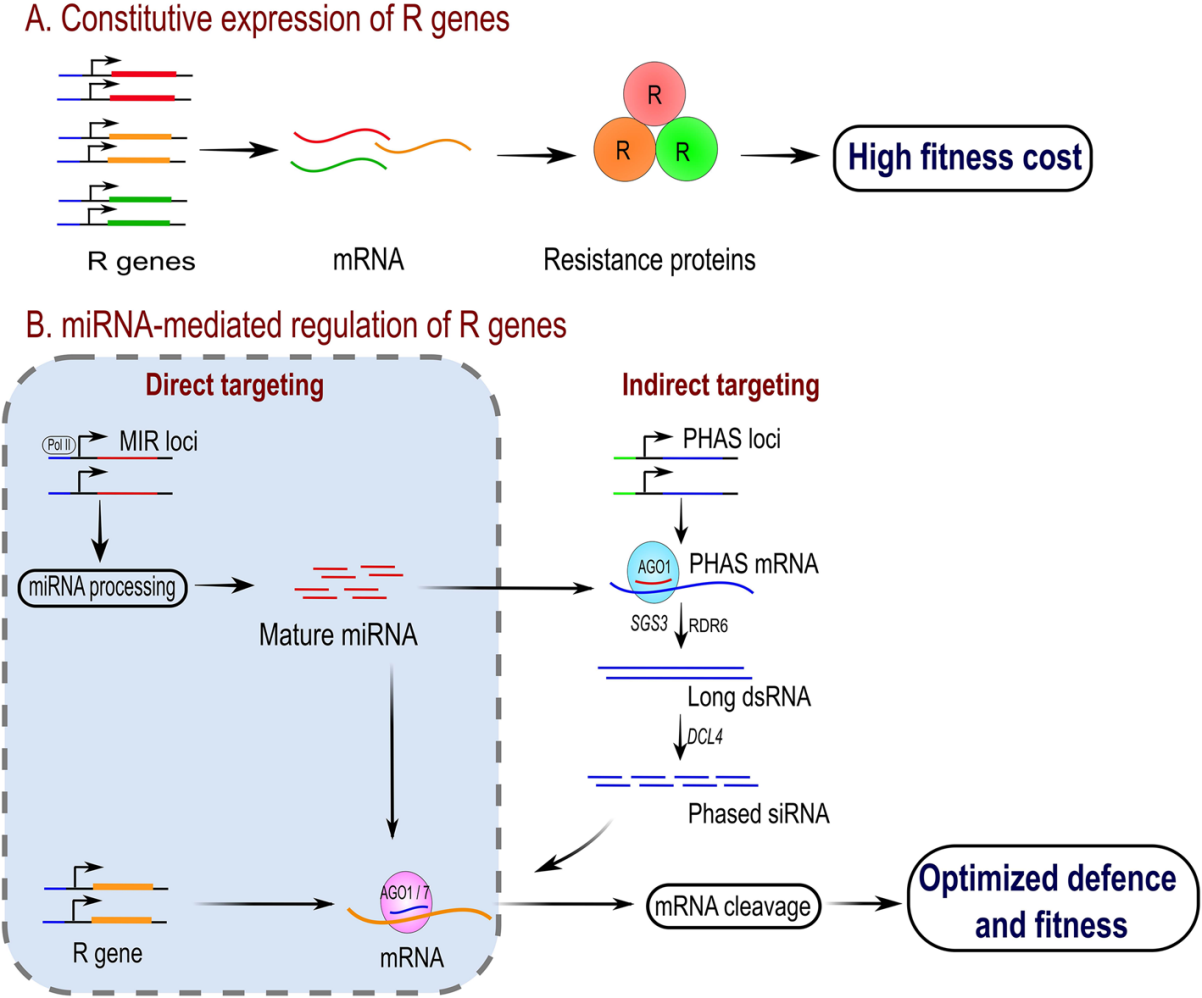
Model depicting the possible roles of miRNAs and phased secondary siRNAs (phasiRNA) in the regulation of R-genes. **A** Under normal conditions, when the host plant is not challenged, constitutive and unregulated expression of R-genes results in high fitness costs; **B** In the direct targeting pathway of R-genes by miRNA, the *MIR* loci produce miRNA transcripts that are processed to mature miRNAs. Subsequently, mature miRNA is complexed with AGO1/7 and directly binds to R-gene transcript followed by cleavage, resulting in basal resistance response and concomitant effects on fitness cost (box with dashed line). In the indirect targeting pathway of R-genes by miRNAs, the mature miRNA is produced from *MIR* loci and interacts with AGO1. The AGO1-miRNA complex binds to *PHAS* transcripts produced from the coding region of *PHAS* loci and cleaves the PHAS transcripts in a sequence-specific manner. *SGS3* and *RDR6* convert the single-stranded RNA to long double-stranded RNA which is processed by *DCL4* to phased siRNA (phasiRNA). *AGO1/7-*phasiRNA complex cleaves R- gene transcripts and maintain the basal level of R-gene expression to achieve optimized and well-balanced resource usage for defense and maintenance of plant fitness by robust growth and development. R genes-resistance genes; AGO1-ARGONAUTE 1; SGS3-*SUPPRESSOR OF GENE SILENCING 3*; RDR6- RNA-DEPENDENT RNA POLYMERASE 6; DCL4- DICER-LIKE 4

Emerging evidence suggests that miRNAs and secondary siRNA play important roles in the regulation of R-gene function by silencing the immune-response receptors when the host plant is not under attack by the pathogen. This mechanism is important in stabilizing basal transcript levels to limit the fitness costs of an overactive immune response. Plants have evolved specific miRNAs that can target the conserved domains of the R-gene in two major pathways, either by direct targeting of R-gene or indirect targeting of R-gene via phasiRNA (Fig. 3). In the pathway for direct targeting of R-genes, a mature miRNA produced from MIR loci interacts with AGO1 protein. The AGO1-miRNA complex binds and cleaves the R-gene transcripts in a sequence-specific manner and prevents the R-gene mediated autoimmunity in the absence of the pathogen.

The *miR472* was the first to be involved in the direct targeting of the CC-NBS-LRRs domain-containing immune receptor genes in *Arabidopsis* [68]. Since its discovery, many other miRNAs involved in R-gene regulation that optimize the defense-fitness trade-off have been reported. For example, the sequence-specific cleavage of the TIR-NB-LRR immune receptor of *N* gene transcripts that conditions resistance to tobacco mosaic virus (TMV) has been shown to be mediated by *nta-miR6019* and *nta-miR6020* modules in the *Solanaceae* family [69]. The *miR482/2118* super-family targeting NB-LRR genes have also been well characterized in tomato, and its prime importance in the regulation of immune response is well documented [70]. A recent report suggested that the *miR1885* is involved in a dynamic balance between plant growth and immunity to viral pathogen in *Brassica* by direct silencing the R-gene [71]. More recently, in rice, *Osa-miR1876* has been shown to epigenetically regulate the expression of the *NBS8R* gene encoding an NB-ARC domain protein that confers resistance to *X. oryzae* [72].

Besides the direct targeting of NLRs, miRNAs have also been shown to indirectly regulate R-genes by targeting other genes that are part of the R-gene networks [73]. For example, the NBS-LRR genes, which are not primarily recognized by miRNA, trigger the production of a phased array (in a sequential, head-to-tail manner, according to the miRNA cleavage site) of 21-nt secondary small interfering RNAs (phasiRNAs) to amplify the silencing effects [74]. These phasiRNAs act in *trans* to silence the addition of R-gene transcripts. In rice, no phasiRNA that regulates R-genes has so far been characterized. However, phasiRNAs regulating reproductive development has been reported.

The phasiRNA targeting R-genes remains unknown in rice. The phasiRNA that targets the conserved motifs of CC-NBS-LRR has been explored in diverse plant species, including spruce, grapevine, poplar, cotton, A*rabidopsis*, and citrus [75, 76]. General observations indicate that the highly conserved siRNAs may be important in optimizing the expression of NBS-LRR genes, which may compromise plant fitness. However, the precise role of phasiRNA in regulating NBS-LRR immune receptors in rice is yet to be fully understood [77]. Furthermore, Fine-tuning of NBS-LRR protein expression by phasiRNA inhibits the constitutive expression of many other R-genes, which could potentially be detrimental to plant growth, leading to trade-offs to productivity when not regulated optimally. In the model legume *Medicago truncatula,* 22-nt miRNAs including *miR2275*, *miR2109*, and *miR2118* have been shown to trigger the production of phasiRNA that are specifically associated with the regulation of other genes encoding NBS-LRR immune receptors [77, 78]. These miRNAs are highly conserved in both legumes and non-legume plant species.

### MicroRNAs fine-tune immunity and trade-offs to yield

High-yielding rice cultivars with resistance to multiple pathogens and pests are paramount to sustainable production. With major accomplishments by breeding, the caveat appeared to be that strong defense responses often come with unintended trade-offs in terms of significant losses to yield as the photosynthetic source-sink dynamics that should favor plant growth tend to be diverted towards defense-related processes [13]. Modern approaches in plant breeding such as marker-assisted selection, transgenics, and genome-editing are being applied to develop resistant cultivars with the minimal penalty to yield. In the past few decades, many R-genes in rice have been tagged with molecular markers for efficient selection and use in pyramiding. However, a limited set of genes are deployed in crop improvement programs since many of them exhibit pleiotropic effects, unwanted linkage drags that undermine agronomic traits, and low heritability [16]. Furthermore, because of the rapid breakdown of R-gene-mediated mechanisms along with their associated fitness costs, the potential use of miRNAs with major roles in regulating the immunity of host rice plants has gained more interest as a more effective means to develop resistant cultivars with optimal balance between resistance and trade-offs to plant vegetative and reproductive growth.

Recent reports have shown that many miRNAs facilitate the maintenance of fitness and resistance with minimal or no penalty to yield. General observations indicate that miRNAs enhance the immunity to pathogens and herbivores with significant positive effects on yield maintenance in most cases. For example, the *Osa-miR159, Osa-miR162, Osa-miR396*, *Osa-miR530*, *Osa-miR1432,* and *Osa-1871* have been shown to have positive effects on yield component traits even when the plant is challenged by pathogens [24, 26, 32, 36–38]. For instance, the miRNA, *Osa-miR156,* fine-tunes resistance without significant penalty to yield, while *Osa-miR1873* confers resistance with only a minor penalty to yield [23, 39].

The *Osa-miR162* synergizes the mechanisms involved in resistance to blast with the genetic potential for yield through the *OsDCL1* that causes the accumulation of intracellular H_2_O_2._ Nevertheless, overexpression of *Osa-miR162* showed significantly narrower grains, lower seed weight, and poor seed set, leading to a significant penalty to grain yield. Silencing of *Osa-miR162* showed positive effects on yield by increasing the number of grains per panicle during *M. oryzae* infection [26]. Similarly, blocking the expression of *Osa-miR530* induces early flowering and seed maturation, with positive effects on grain number per panicle and grain weight. The underlying regulatory networks that balance yield-related processes and immunity is an area that is currently under investigation [36].

Likewise, downregulation of *Osa-miR1432* enhances both immunity and yield by targeting the *OsEFH1* protein [37]. The resistance and yield trade-off are also fine-tuned by the *miR1871*-*MFAP1* module. Transgenic rice overexpressing *Osa-miR1871* as well as the *mfap1* mutants exhibit significant reductions in grain yield. Conversely, downregulation of *Osa-miR1871* displayed the opposite effects. These results suggested that *Osa-miR1871* regulates yield-related traits and immunity through the function of *MFAP1* [38]. Suppression of *Osa-miR396* induces multiple *growth-regulating factor* (*GRF)* genes that enhance resistance to blast and BPH [32, 52]. The *Osa-miR396* also plays important roles in the regulation of cellular processes that are critical in the maintenance of inherent potentials for grain size, grain yield, inflorescence development, panicle branching, as well as tolerance to saline and alkaline soil and water [79–81].

The *Osa-miR156* negatively regulates host immunity against blast, bacterial blight, and BPH by targeting the *IPA1* (*Ideal Plant Architecture 1)*, *OsSPLs (SQUAMOSA Promoter-binding protein-like transcription factors),* and several *OsWRKY* genes. Sequestering *Osa-miR156* by target mimicry led to enhanced resistance to all three biotic stressors by affecting the accumulation of target transcripts, including the products of defense-related genes [23, 41, 51]. However, overexpression of modified *OsSPL14* promotes the panicle branching, which leads to improvements in the number of spikelets per panicle, stronger culm, and tiller reduction. These effects contributed to a significant enhancement of grain yield [82, 83].

Transgenic studies showed that inducible expression of the *IPA1* gene under the control of bacterial effector-induced promoter leads to enhanced resistance to BB, with concomitant improvement in grain size, plant architecture, and grain yield [41]. The *Osa-miR1873* has been shown to balance the cellular processes involved in the expression of resistance to blast and maintenance of plant growth. Overexpression of *Osa-miR1873* has been shown to compromise resistance with additional negative effects to plant growth and developments as manifested by a significant reduction in yield potential due to the reduction of seed-set as indicated by the proportion of filled grains per panicle. In contrast, studies that sequestered *Osa-miR1873* showed that proper regulation causes no significant effects on yield [39]. Other miRNAs such as *Osa-miR159*, *Osa-miR160, Osa-miR164, Osa-miR167*, and *Osa-miR398* have been shown to affect different traits that are a critical component of yield potential, independently.

### MicroRNAs coordinate immune response with other cellular processes that determine agronomic potential

MicroRNAs rarely work independently. Single miRNA-encoding loci often function in the regulation of multiple cellular processes, hence more than one trait. Recent studies in rice showed that the role of miRNAs is not limited to the regulation of defense response mechanisms, but they are also involved in the regulation of complex traits (Table 2). For example, the *Osa-miR156* is known to regulate a total of eleven (11) *SPL* genes involved in diverse biological and developmental processes in rice. The *Ideal Plant Architecture 1*, which encodes a *SQUAMOSA Promoter-binding protein-like transcription factors* is known to be targeted by *Osa-miR156*. Overexpression of *OsSPL14* enhanced resistance to bacterial blight accompanied by a substantial improvement in yield caused by the reduction in the number of unproductive tillers and enhancement of panicle branching [41]. Mutation in *OsSPL14* perturbs *Osa-miR156*-mediated regulation of *OsSPL14,* leading to positive gains in yield maintenance as indicated by the reduction of unproductive tillers and stronger culm [82]. Furthermore, overexpression of *OsSPL14* also enhanced the number of grains per panicle by increasing the panicle branching [83].

**Table 2:** List of known microRNAs involved in the integration of defense-related responses with growth and development-related responses

miRNAs	Biotic stresses	Traits	References
*Osa-miR156*	Blast, BB, BPH	Grain size, grain yield, grain quality, panicle branching, Seed germination, tillering, plant architecture	[82–88]
*Osa-miR159*	Blast, BB	Floral development, stem elongation, leaf development, grain size	[89–91]
*Osa-miR160*	Blast	Rice growth and development, tillering, seed setting rate	[92, 93]
*Osa-miR162*	Blast	Drought tolerance, plant development	[94, 95]
*Osa-miR164*	Blast, BB	Drought tolerance, plant architecture, grain yield	[96–98]
*Osa-miR166*	Blast	Nutrient ion uptake, drought tolerance, cadmium tolerance	[99, 100]
*Osa-miR167*	Blast, BB	Auxin response, tiller number, grain weight	[101–103]
*Osa-miR169*	Blast, BB	Nitrogen-use efficiency, salt stress	[43, 104]
*Osa-miR319*	Blast, RRSV	Leaf morphogenesis, cold tolerance, plant height	[105–107]
*Osa-miR396*	Blast, BPH, Foot rot	Grain size, grain yield, floral development, stem elongation, salt, and alkali tolerance	[79, 80, 87, 108–112]
*Osa-miR398*	Blast	Panicle length, grain number, grain size, abiotic stress	[88, 113]
*Osa-miR444*	Blast, RSV	Tillering, nitrate signalling, root development	[114–116]
*Osa-miR528*	RSV	Flowering time, pollen development, arsenite tolerance, cold tolerance	[117–120]

*BB* Bacterial blight, *BPH* Brown planthopper, *RRSV* Rice ragged stunt virus, *RSV* Rice stripe virus

It was recently shown that downregulation of *Osa-miR156fhl-3p* enhances host plant immunity to *M. oryzae* by positively affecting *OsSPL14* and *WRKY45* [23]. Furthermore, recent reports also showed that *Panicle blast 1* (*Pb1*), a panicle blast resistance gene encoding a coiled-coil, nucleotide-binding site, leucine-rich repeat (CC-NBS-LRR) protein, interacts with a WRKY45 transcription factor, which plays a critical role in the expression of induced resistance through the salicylic acid signaling pathway regulated by the ubiquitin-proteasome system [121]. Evidence also supports that suppression of *Osa-miR156* could promote seed dormancy by inducing the expression of *OsSPL14* and repressing the GA pathway [84]. Expression of *OsSPL13* promotes yield in rice by improving grain size and panicle length [122]. The *OsSPL16* improves grain size by binding to *GW7* [85, 123]. The *OsSPL18* binds to the promoter of *DEP1* and negatively regulates the cellular process for grain number potential [124, 125]. Overexpression of *OsSPL7* negatively affects tiller number and positively affects plant height [86]. In rice, the *OsSPL9* is involved in the regulation of *Osa-miR528*, which promotes flowering under long-day conditions by repressing the *Red and Far-red Insensitive 2* (*OsRFI2*) gene [119]. The *OsSPL9* regulates the *Osa-miR528/L-Ascorbate Oxidase (AO)* transcriptional module, which enhances anti-viral defenses [50]. Moreover, *Osa-miR528* regulates pollen intine formation by targeting the uclacyanin gene *OsUCL23* [120]. Likewise, in creeping bentgrass, it has been shown that constitutive expression of *Osa-miR528* enhances tolerance to salinity and nitrogen-starvation [126].

Hormones are the foundations of cellular signaling that integrate growth-related and defense-related responses [11]. In rice, genes involved in auxin signaling particularly the *auxin response factors* (ARFs) are known to be targeted by *Osa-miR160* and *Osa-miR167*. The *Osa-miR160* is known to positively regulate blast resistance, whereas *Osa-miR167* acts in an antagonistic fashion. Furthermore, alteration in auxin signaling in rice through the upregulation of *Osa-miR160-resistant OsARF18* has been shown to cause severe defects in overall plant growth as well as reproductive development, with negative effects on seed size and seed set [92, 93]. The *Osa-miR167* promotes an efficient response to auxin, which translates to the concomitant enhancement of tiller number and grain weight [101–103]. The *growth regulating factors* (*GRFs*) targeted by *Osa-miR396* regulates diverse biological processes. It has been shown that manipulating the *Osa-miR396-OsGRFs* module substantially increases grain size and yield. Specifically, mutation of the *OsGRF4* (*GS2*) perturbs the function of *Osa-miR396* and its regulatory role over *OsGRF4* [79, 87, 108]. Additionally, *Osa-miR396* promotes panicle branching by regulating *OsGRF6* [80]*.*

Several immune-responsive miRNAs play a critical role in regulating cellular processes that determine nutrient uptake as well as responses to other types of abiotic stresses. For example, the *Osa-miR164*, which is important in processes that control grain yield and other plant architecture traits in rice, has been shown to affect drought tolerance [96, 97]. The *Osa-miR166* is involved in the regulation of cellular processes determining nutrient and ion uptake, drought tolerance, and cadmium tolerance [99, 100, 127]. The pathogen–responsive *Osa-miR169* has been shown to regulate nitrogen uptake and salinity tolerance in rice [43, 104]. The *Osa-miR319* regulates leaf morphogenesis, plant height, and improves cold tolerance [105–107]. The *Osa-miR398* not only modulates panicle length, grain number, and grain size but also regulates various abiotic stresses [88, 113]. The *Osa-miR444* modulates MADS-box transcription factors to regulate tillering and nitrate signaling [114–116]. The RSV-responsive *Osa-miR528* regulates tolerance to arsenite and low temperature in rice [117–120].

### Manipulation of miRNAs as a novel approach for the development of high-yielding disease and insect-resistant rice cultivars

Knock-out mutation of miRNAs is an important approach to validating cellular functions through the identification of their downstream target genes. Furthermore, miRNAs belong to conserved families comprised of multiple members with potentially redundant functions. Therefore, loss-of-function analysis of miRNA-encoding loci has become a major challenge. Traditional methods of mutagenesis (chemical/radiation/T-DNA insertion) are largely ineffective due to the small size of miRNA molecules. Currently, target mimics (TMs), short tandem target mimics (STTMs), molecular sponges (SPs), and artificial miRNAs (amiRNAs) are the most commonly used techniques for loss-of-function analysis of miRNAs. Among them, the miRNA decoy techniques such as TMs and STTMs have been widely adopted for functional analysis [128]. TMs and STTMs efficiently suppress the endogenous activity of highly abundant miRNA molecules and nullify the suppression effects to the target gene. This in turn changes the phenotype through the accumulation of target transcripts. However, neither of these approaches provides the most efficient silencing effects to the endogenous activity of the cognate miRNA genes.

Genome editing technologies such as zinc finger nuclease (ZFN), transcription activator-like effector nucleases (TALEN), and clustered regularly interspaced short palindromic repeats (CRISPR)-based system are the more recent cutting-edge approaches for the control of endogenous miRNA abundance [129]. These strategies work based on the principle of binding an exonuclease to a target region in the genome, creating a double-stranded break (DSB). To stabilize the genome, these breaks are repaired by one of two methods: Non-Homologous End-Joining (NHEJ) and Homology Directed Repair (HDR), which causes insertions and deletions (indels) or incorporation of a larger sequence at the repair site. When these changes occur within the miRNA coding region, they can reduce the rate of miRNA biogenesis leading to incomplete loss of function or production of new miRNAs. In contrast, large deletions can cause a complete loss of function of specific miRNAs by producing null mutants. Editing the target gene also hinders miRNA activity due to errors in binding.

Zinc-finger nucleases (ZFNs) are artificial restriction enzymes generated by fusing zinc-finger-based DNA-recognition modules with the DNA-cleavage domain. Each zinc finger typically recognizes and binds to a nucleotide triplet, and fingers are often assembled into groups to bind to specific DNA sequences. TALENS is equipped with the same tenet as ZFN with a target specificity from the protein-DNA association. In addition, it can recognize a single nucleotide using specific amino acid repeats to recognize a single nucleotide, thus providing more choices for selecting the target locus. CRISPR-mediated genome editing has revolutionized nuclease targeting [130–133]. In the CRISPR-Cas system, a single guide RNA (sgRNA) or double guide-RNA (dgRNAs) recognizes the target sequence, and the double-strand break caused by Cas9 (CRISPR associated protein 9) or Cpf1(CRISPR from Prevotella and Francisella 1) nuclease, induces insertion or deletion through the process of DNA repair. The CRISPR-based system has higher efficacy and efficiency than the other methods.

Recently, CRISPR/Cas9 was efficiently used in rice to introduce heritable mutations in mature miRNAs [134–136]. Knockout mutations in *Osa-miR396e* and *Osa-miR396f* using the CRISPR/Cas9 system have been shown to create genetic gains through the enhancing effects to yield component traits such as grain size and panicle branching, particularly under nitrogen deficit conditions [81]. CRISPR/Cas9-mediated mutagenesis of *Osa-miR396ef* has been shown to promote the efficiency of GA signaling, with concomitant enhancement in grain yield contributed by improvements in grain size, leaf blade and sheath anatomy and morphology [112]. Targeted mutagenesis of a single miRNA locus (e.g.*, Osa-miR408, Osa-miR528*) or entire miRNA gene families (e.g.*, Osa-miR815, Osa-miR820*) has been achieved by a CRISPR/Cas9 mediated strategy in rice. It has been shown that under salinity stress, larger deletion in *Osa-miR528* led to elevated transcript levels from the target genes [134]*.* Similarly, targeted mutagenesis of *OsSPL9* using CRISPR)/Cas9 led to a significant reduction in *Osa-miR528* expression, suggesting a critical role of *OsSPL9* in transcriptional regulation of *Osa-miR528* [119].

Furthermore, a series of CRISPR/Cas9-mediated mutations in *Osa-miR156* have been investigated for their effects on seed dormancy [84]. CRISPR/Cas9 system has also been employed to introduce mutations in multiple *superoxide dismutase* (*SOD*) genes to examine their effects on *Osa-miR398*-mediated resistance to *M. oryzae* [33]. Targeted mutations were introduced in the target gene (*OsARF12*) of *Osa-miR167d* using the CRISPR/Cas9 method to investigate *Osa-miR167-ARF12* interaction in immunity against rice blast disease [29].

### Conclusion and future directions

Continuously emerging evidence from various experimental systems and approaches established the essentiality of miRNA-mediated regulatory mechanisms in integrating defense-related responses to fungal, bacterial, and viral pathogens, and insect herbivores, with growth and development-related responses. MicroRNA regulatory mechanisms have direct roles in host plant immunity by directly suppressing target genes with either negative or positive effects, or by indirectly inducing signaling pathways with different classes of phytohormones and other small molecules such as ROS. Genomics-enabled biology offers an excellent opportunity to identify more miRNAs involved in fine-tuning resistance to biotic stresses to minimize trade-offs to productivity. Harnessing these miRNAs could be useful in breeding disease and pest-resistant rice cultivars without the drags to the agronomic potential that are typically observed when manipulating R-genes alone.

Identifying new miRNAs associated with defense mechanisms against pests and diseases could be achieved by employing high-throughput sequencing technologies. Specifically, this approach involves the identification of differentially expressed miRNAs under stress via miRNA-Seq followed by in silico analysis and degradome sequencing to predict miRNA targets. Once the specific roles of the miRNAs and target genes are identified, it would be possible to engineer them through genome-editing technologies. Site-specific mutagenesis of target genes impairs the cleavage by miRNA, which allows the development of transgenic plants with disease and insect resistance. In addition to functional analysis, miRNAs are usually conserved in nature, hence limited sequence diversity. The miRNA binds to their target genes in a sequence-specific manner. Variation in miRNAs leads to a variable level of transcript accumulation that causes compounded phenotypes.

## Data Availability

Data sharing not applicable to this article as no datasets were generated or analyzed during the current study.

## Abbreviations

PAMPs: Pathogen-associated molecular patterns
DAMPs: Damage-associated molecular patterns
PRRs: Pattern-recognition receptors
PTI: PAMP-triggered immunity
ETS: Effector-triggered susceptibility
ETI: Effector-triggered immunity
HR: Hypersensitive response
DCL1: DICER-like 1
ROS: Reactive oxygen species
STTM: Short tandem target mimic
ARF: Auxin response factor
IAA: Indole-3-acetic acid
EIN2: Ethylene insensitive 2
SOD: Superoxide dismutase
Nramp 6: Natural resistance-associated macrophage protein 6
SPL: SQUAMOSA promoter-binding protein-like transcription factor
NF-YA: Nuclear factor Y-A
DHFR-TS: Dihydrofolate reductase/thymidylate synthase
HPODE: Hydroperoxy-octadecadienoic acid
GRFs: Growth-regulating factors
TCP: TEOSINTE BRANCHED, cycloidea and PCF
ZFN: Zinc finger nuclease
TALEN: Transcription activator-like effector nucleases
CRISPR: Clustered regularly interspaced short palindromic repeats
NHEJ: Non-homologous end-joining
HDR: Homology-directed repair
R-genes: Resistance genes
AGO1: Argonaute 1
SGS3: Suppressor of gene silencing 3
RDR6: RNA-dependent RNA polymerase 6
RSV: Rice stripe virus
RRSV: Rice ragged stunt virus
